# Beyond iodine nutrition: socioeconomic patterns independently drive thyroid disease risk in pregnant women in Xinjiang

**DOI:** 10.3389/fnut.2026.1789303

**Published:** 2026-06-19

**Authors:** Jing Zhou, Yujuan Fan, Rishalaiti Tayier, Dannier Abuduwaili, Dawureni Muhetaer, Chenchen Wang, Shunhua Wu

**Affiliations:** 1 School of Public Health, Xinjiang Medical University, Urumqi, China; 2 Health Hazard Factor Detection and Control Institute, Xinjiang Uygur Autonomous Region Center for Disease Control and Prevention, Urumqi, China; 3 Xinjiang Institute of Socialism, Urumqi, China

**Keywords:** iodine nutrition, latent class analysis, mediation analysis models, pregnancy, socioeconomic status, thyroid diseases

## Abstract

**Objective:**

To investigate the impact of distinct socioeconomic status (SES) patterns on the risk of thyroid diseases in pregnant women against the background of adequate iodine nutrition.

**Methods:**

A cross-sectional study was conducted in Xinjiang from 2021 to 2022, enrolling 1,166 pregnant women. Fasting venous blood and urine samples were collected to assess thyroid function and iodine nutritional indicators. Long-term and short-term iodine intake were evaluated using a food frequency questionnaire and a 3-day 24-h dietary recall, respectively. Latent class analysis (LCA) was used to identify socioeconomic-environmental patterns based on region, income, education, occupation, and passive smoking status. Multivariable regression models were constructed to analyze the association between SES patterns and thyroid outcomes, adjusting for maternal and environmental covariates. Mediation analysis models were used to assess the mediating effect of iodine nutritional indicators.

**Results:**

Three SES patterns were identified: Pattern A (Northern Xinjiang-Housewife-dominant Low SES, 37.14%), Pattern B (Southern Xinjiang-Housewife-dominant Low-to-Middle SES, 41.60%), and Pattern C (Highly Educated-Professional Women-High SES, 21.27%). Compared with Pattern A, Pattern C was significantly associated with lower TSH levels (*β* = −0.17, 95% CI: −0.32 – −0.03, *p* = 0.019). In the logistic regression models, Pattern B was significantly associated with lower odds of thyroid nodules compared with Pattern A (OR = 0.32, 95% CI: 0.14–0.74, *p* = 0.008). No statistically significant associations were observed between SES patterns and other thyroid disease outcomes after adjustment. Mediation analysis suggested that although dietary iodine intake and urinary iodine-to-creatinine ratio differed across SES patterns, these iodine nutritional indicators did not show statistically significant mediation in the associations between SES patterns and thyroid-related outcomes (*p* > 0.05).

**Conclusion:**

Against the overall background of adequate iodine nutrition, differences in socioeconomic patterns were independently associated with thyroid disease outcomes in pregnant women in Xinjiang, and this influence is not dependent on differences in iodine intake. This study suggests that prevention and control strategies for perinatal thyroid diseases should extend beyond single nutritional interventions towards psychological support and comprehensive environmental management targeting specific social roles.

## 1 Introduction

Thyroid health during pregnancy is critical not only for maternal well-being but also for the early neurological development of the fetus and pregnancy outcomes (1). Previous studies have demonstrated significant associations between thyroid dysfunction during gestation and miscarriage, preterm birth, hypertensive disorders of pregnancy, and impaired neurocognitive development in offspring (2–4). Consequently, identifying high-risk factors for gestational thyroid disorders and implementing early interventions are key priorities in perinatal medicine and public health.

Iodine, an essential trace element for thyroid hormone synthesis, has long been considered the primary environmental factor influencing thyroid health (5). Xinjiang was once one of the most severely iodine-deficient regions in China (6). Due to the barrier effect of the Tianshan Mountains, significant natural and social heterogeneity exists between Northern and Southern Xinjiang in terms of geographical environment, economic structure, population composition, and dietary habits (7). Although the long-term implementation of universal salt iodization has enabled Xinjiang to achieve the goal of eliminating iodine deficiency disorders at an aggregate level, this average-level attainment may obscure health disparities within specific subpopulations (8). Epidemiological surveys have revealed significant variations in the prevalence of thyroid diseases among different populations, even against a backdrop of adequate iodine nutrition (9–11). This phenomenon suggests that, beyond iodine intake as a single factor, more subtle social and environmental factors may be driving the uneven distribution of thyroid health.

Socioeconomic status (SES), as a social determinant of health, influences health outcomes through multiple pathways, including material living conditions, psychosocial stress, lifestyle, and healthcare utilization (12, 13). Traditional epidemiological studies often analyze the health impact of income, education, or occupation as single variables; however, such approaches fail to comprehensively capture an individual’s true position within a complex social structure (14–16). Therefore, it remains a question worthy of in-depth investigation whether the disparities in thyroid health among different populations are primarily mediated by differences in dietary iodine intake or are more attributable to social contextual factors beyond nutrition. Clarifying this etiological factor is decisive for health policy formulation, as it directly determines whether prevention and control strategies should focus solely on nutritional supplementation or on broader social support. Based on this rationale, the present study, grounded in the context of Xinjiang, employs a person-centered Latent Class Analysis (LCA) model to identify the authentic socioeconomic profiles within the pregnant population. Combined with causal inference methods, this study aims to investigate whether differences in socioeconomic patterns independently drive the risk of thyroid disorders among pregnant women in Xinjiang, beyond iodine nutritional status. The findings are expected to provide a scientific basis for breaking the intergenerational transmission of health inequality and for developing differentiated, precise prevention and control strategies for perinatal thyroid disorders in the Xinjiang region.

## 2 Materials and methods

### 2.1 Study design and participants

The unique geographical barrier formed by the Tianshan Mountains in Xinjiang leads to marked differences in climate, natural environment, and dietary patterns between northern and southern Xinjiang. This cross-sectional study was conducted from 2021 to 2022 as part of the special iodine nutrition survey among pregnant women in Xinjiang. The primary objective of this study was to investigate the association between socioeconomic status patterns, iodine nutritional status, and thyroid health among pregnant women in northern and southern Xinjiang. Pregnant women were recruited by the local Centers for Disease Control and Prevention from selected survey sites in Ili Kazakh Autonomous Prefecture, representing northern Xinjiang, and Aksu Prefecture, representing southern Xinjiang. The sampling frame consisted of pregnant women who were registered in the local maternal health care system and attended routine antenatal care at the selected survey sites during the study period. Eligible pregnant women were consecutively invited to participate according to the predefined inclusion and exclusion criteria. The inclusion criteria were as follows: (1) age ≥18 years; (2) residence in the local area for more than 3 years; (3) current pregnancy; (4) ability to complete the questionnaire survey; (5) willingness to provide urine and blood samples; and (6) provision of written informed consent. The exclusion criteria were as follows: (1) history of thyroid disease before pregnancy, including hyperthyroidism, hypothyroidism, thyroiditis, thyroid surgery, or thyroid nodules requiring treatment; (2) use of thyroid-related medications, iodine-containing medications, or iodine supplements other than routine iodized salt; (3) severe chronic diseases that could affect iodine metabolism or thyroid function, such as chronic kidney disease, severe liver disease, autoimmune diseases, or malignant tumors; (4) special dietary patterns that could substantially affect iodine intake; (5) incomplete questionnaire information; or (6) missing biological samples. Participants were classified into early pregnancy (<13 gestational weeks), mid-pregnancy (13–27 gestational weeks), and late pregnancy (≥28 gestational weeks). All participants provided written informed consent after being fully informed of the study details. This study was approved by the Ethics Committee of the Xinjiang Uygur Autonomous Region Center for Disease Control and Prevention (Approval No.: 2021-07). The study protocol strictly adhered to the relevant principles of the Ethical Review Measures for Biomedical Research Involving Humans and the Declaration of Helsinki. All participants provided written informed consent after being fully informed of the study details.

### 2.2 Questionnaire survey

The questionnaire survey was conducted at enrollment and consisted of three parts: a basic information questionnaire, a food frequency questionnaire (FFQ), and a 3-day 24-h dietary recall questionnaire. The gestational week and trimester at the time of questionnaire administration were recorded for each participant. The basic information questionnaire collected data on maternal age, gestational week, self-reported height and body weight at enrollment, parity, annual income, education level, occupation, and passive smoking status. BMI at enrollment was calculated as self-reported body weight in kilograms divided by self-reported height in meters squared (kg/m^2^). To assess long-term dietary iodine intake patterns during pregnancy, a locally validated FFQ was administered at enrollment. Participants were asked to retrospectively report the frequency and portion sizes of common foods and beverages consumed during the 3 months before the survey. It specifically included food varieties commonly consumed in Xinjiang, with particular attention to major dietary sources of iodine, including iodized salt, seafood, eggs, milk and dairy products, and iodine-containing condiments. To obtain more precise data reflecting recent dietary iodine intake, a retrospective 3-day 24-h dietary recall, including two weekdays and one weekend day, was conducted for all participants at enrollment. Participants were asked to recall in detail the types and quantities of all foods, beverages, and condiments consumed during the selected 3 days before the survey, with the aid of standard portion size pictures for estimation.

### 2.3 Sample collection and testing

#### 2.3.1 Food sample collection and testing

Based on the results from the FFQ and 3-day 24-h dietary recall, all major food categories routinely consumed by the participants were systematically collected from representative large-scale markets, supermarkets, farmers’ markets, and grocery stores in their residential areas. After uniform pre-treatment and digestion, the iodine content of all food samples was determined using inductively coupled plasma mass spectrometry (ICP-MS; Thermo Fisher Scientific, iCAP Q) (17).

#### 2.3.2 Biological sample collection and testing

Immediately after completion of the questionnaire survey on the day of enrollment, biological samples were collected from each pregnant woman by trained healthcare professionals. A 5-mL random midstream urine sample and a 4-mL fasting venous blood sample were collected from each participant. The gestational week and trimester at the time of biological sample collection were therefore consistent with those recorded at questionnaire administration. The urinary iodine concentration was determined by ICP-MS (18). Urinary creatinine levels were measured using a fully automated biochemical analyzer (Roche Diagnostics, cobas c701), and the urinary iodine-to-creatinine ratio (UICr) was calculated to correct for the influence of urine concentration or dilution on the urinary iodine determination results (19). After centrifugation of the blood samples to separate the serum, thyroid function was assessed by measuring five parameters—free triiodothyronine (FT3), free thyroxine (FT4), thyroid-stimulating hormone (TSH), thyroglobulin antibody (TgAb), and thyroid peroxidase antibody (TPOAb)—using a fully automated electrochemiluminescence immunoassay analyzer (Roche Diagnostics, cobas e411).

#### 2.3.3 Thyroid ultrasound examination

Standardized thyroid ultrasound examinations were performed on all study subjects by the same senior sonographer with over ten years of clinical experience, using a high-frequency linear array probe ultrasound system (Samsung Medison, Sonoace R3) calibrated daily. During the examination, the pregnant women were placed in a supine position with the neck moderately extended. The sonographer systematically measured and recorded the length (L), width (W), and thickness (D) of the left and right thyroid lobes. Each measurement was repeated three times, and the average value was used to calculate the thyroid volume. Concurrently, the number, size, margin, morphology, and echogenicity of any thyroid nodules were carefully observed and documented. This unified and professional operational protocol was designed to maximize the objectivity and comparability of the data, effectively minimizing inter-observer variation bias.

### 2.4 Calculation of related indicators and diagnostic criteria

#### 2.4.1 Calculation of dietary iodine intake

Both the FFQ and the 3-day 24-h dietary recall were used to estimate average dietary iodine intake, reflecting long-term patterns, and daily dietary iodine intake, reflecting recent precise status, respectively (20, 21). The formula for calculating dietary iodine intake based on the FFQ is as follows:

$$I_{i}=F_{i}\cdot P_{i}\cdot C_{i}$$

Where *C_i_* represents the measured iodine content of that food category; *F_i_* denotes the average daily intake frequency of that food category; and *P_i_* indicates the average single-serving portion size of that food category. First, the average daily intake frequency and average single-serving portion size for each iodine-containing food item were extracted from the questionnaire to calculate the daily iodine intake from individual foods. Subsequently, the daily iodine intake values *I_i_* from all iodine-containing foods were summed. The iodine contribution from iodized salt was estimated separately and incorporated into the aforementioned cumulative sum, ultimately yielding the total dietary iodine intake in micrograms per day (μg/d).

The average daily dietary iodine intake based on the 3-day 24-h dietary recall method was calculated by aggregating the iodine intake from various foods, drinking water, and salt consumed by the study participants during the survey period, then dividing by the number of survey days. The specific calculation formula is as follows:

$$D\prod_{24h}=\frac{1}{3}\{\sum_{d=1}^{3}[\sum_{i}(A_{\mathrm{id}}\cdot C_{i})+W_{d}\cdot C_{w}+S_{d}\cdot C_{s}\cdot (1-0.20)]\}$$

Where DII_24h_ represents the average daily iodine intake estimated from the 3-day dietary recall (μg/day); *A_id_* is the amount of food item *i* consumed on day *d*; *C_i_* is the iodine concentration of food item *i*; *W_d_* is the drinking water intake on day *d*; *C_w_* is the iodine concentration in drinking water; *S_d_* is the salt intake on day *d*; *C_s_* is the iodine concentration in salt; and 0.20 represents the assumed 20% iodine loss during cooking. Data on iodine concentrations in drinking water and salt were referenced from the 2021 Xinjiang Iodine Deficiency Disorders Surveillance Report (8). The median iodine concentration in drinking water was 4.80 (1.95, 7.80) μg/L in the Aksu region and 3.70 (1.40, 4.80) μg/L in the Ili region. The median iodine concentration in salt was 26.71 (24.20, 30.18) mg/kg in Aksu and 26.57 (24.85, 28.70) mg/kg in Ili. According to the national standard for edible salt (GB 26878-2011), the iodine content in salt from both regions met the relevant requirements for iodized salt. All food, water, and salt iodine concentrations were converted to consistent units before calculation.

#### 2.4.2 Calculation of thyroid volume

Thyroid volume was calculated based on ultrasound measurements using the formula:

V=0.479·L·W·D

Where *V* represents the volume of one thyroid lobe, *L* represents length, *W* represents width, and *D* represents depth. Total thyroid volume was calculated as the sum of the volumes of the left and right thyroid lobes.

#### 2.4.3 Diagnostic criteria for thyroid function and thyroid diseases

The assessment of thyroid hormone levels was based on standards established for the Roche electrochemiluminescence immunoassay system (22). The appropriate range for FT3 was 3.1–6.8 pmol/L. The normal reference ranges for TSH were 0.09–4.52 mIU/L, 0.45–4.32 mIU/L, and 0.30–4.98 mIU/L for the first, second, and third trimesters, respectively. The normal reference ranges for FT4 were 13.15–20.78 pmol/L, 9.77–18.89 pmol/L, and 9.04–15.22 pmol/L for the corresponding trimesters. TgAb >115 IU/mL and TPOAb >34 IU/mL were defined as positive.

### 2.5 Data processing and statistical analysis

#### 2.5.1 Data preprocessing

All data cleaning and statistical analyses were performed using R software (version 4.3.4). Key R packages included poLCA, lme4, lmerTest, mediation, mice, and boot. Categorical variables were converted to factors. Normality tests and skewness calculations were conducted for continuous variables. Variables with an absolute skewness greater than 1, indicating non-normal distribution, were log-transformed to meet model assumptions. Pregnant women with missing basic information data or lacking biological samples were directly excluded from the dataset. All statistical tests were two-sided, with *p* < 0.05 considered statistically significant.

#### 2.5.2 Latent class analysis

To identify potential socioeconomic and household environmental exposure patterns within the pregnant women population, LCA was performed using five categorical indicators: region, annual household income, education level, occupation before pregnancy, and passive smoking status (23). These indicators were selected to capture both conventional socioeconomic characteristics and household environmental exposure contexts that may be relevant to maternal thyroid health. Region represented the broader geographical and contextual socioeconomic environment; annual household income reflected household economic resources; education level represented individual educational attainment and health literacy; occupation before pregnancy reflected employment status and social role characteristics; and passive smoking status was included as a household environmental exposure indicator that may be socially patterned and potentially associated with maternal health outcomes (24). Region was categorized as northern Xinjiang (Ili Kazakh Autonomous Prefecture) and southern Xinjiang (Aksu Prefecture). Annual household income was coded into five categories: low income (<20,000 yuan/year), below average income (20,000–30,000 yuan/year), average income (30,000–50,000 yuan/year), above average income (50,000–100,000 yuan/year), and high income (>100,000 yuan/year). Education level was coded as primary or below, junior high, high school and college or above. Occupation before pregnancy was coded as housewife, worker, and farmer. Passive smoking status was coded as no exposure or exposure to second-hand smoke. Models with 2 to 5 classes were fitted sequentially. The final number of latent classes was determined by comprehensively considering the Akaike Information Criterion (AIC), Bayesian Information Criterion (BIC), entropy, model interpretability, and the minimum class proportion. The identified SES patterns were named and qualitatively described based on the conditional probability distribution of each indicator across different classes.

#### 2.5.3 Multivariable regression analysis

Multivariable linear and logistic regression models were employed to assess the association between SES patterns and thyroid health outcomes. To control for regional differences between Northern and Southern Xinjiang, region was included as a fixed-effect covariate in all models. Given the limited number of hierarchical levels (*n* = 2), a fixed-effect approach was prioritized over a random-intercept model to ensure more reliable parameter estimation and avoid instability in variance component estimation. Outcome measures included continuous variables (FT3, FT4, TSH, and thyroid volume) and binary variables (thyroid nodules, thyroid antibody positivity, hypothyroidism, hyperthyroidism, and isolated hypothyroxinemia). For continuous outcomes, multivariable linear regression was used, reporting *β* coefficients and 95% confidence intervals (CIs). For binary outcomes, multivariable logistic regression was used, reporting odds ratios (ORs) and 95% CIs. All regression models were adjusted for maternal age, BMI, gestational age, parity, region, and passive smoking exposure. Due to the complete absence of variance (0% prevalence), active smoking and alcohol consumption were not included as covariates in the multivariable models, as statistical adjustment for these invariant factors is mathematically non-feasible.

#### 2.5.4 Mediation effect analysis

To explore the underlying mechanisms through which SES patterns affect thyroid health, this study examined whether iodine nutrition indicators (average dietary iodine intake, daily dietary iodine intake, and UICr) served as mediating variables. The average causal mediation effect (ACME), average direct effect (ADE), total effect, and proportion mediated were calculated using a quasi-Bayesian Monte Carlo simulation method with 1,000 iterations. For binary outcomes, the mediation analysis model employed a Probit link function to ensure estimation accuracy (25).

## 3 Results

### 3.1 Identification of latent classes of socioeconomic status

**Figure 1:**
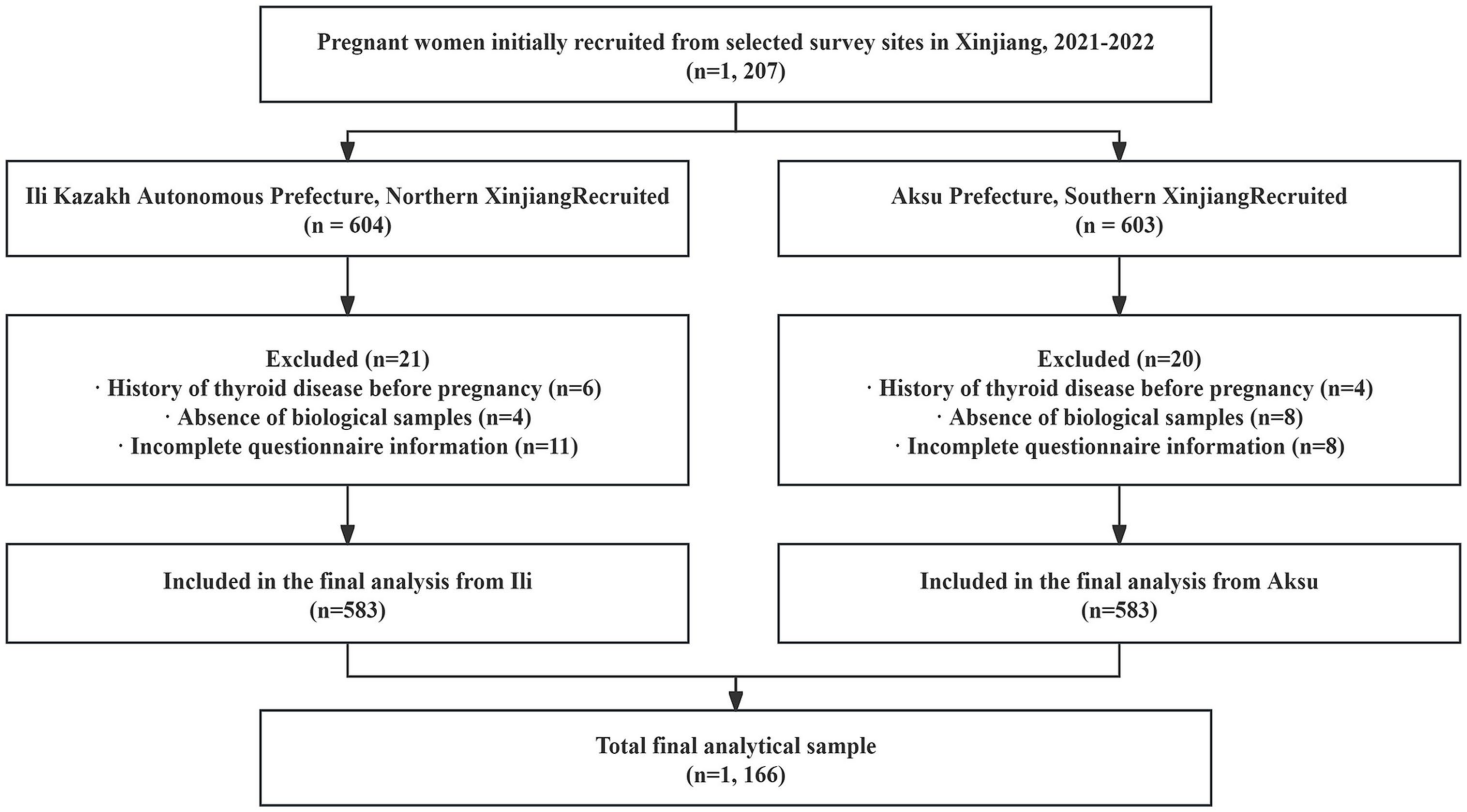
Flowchart of participant recruitment, screening, exclusion, and final inclusion.

**Figure 2:**
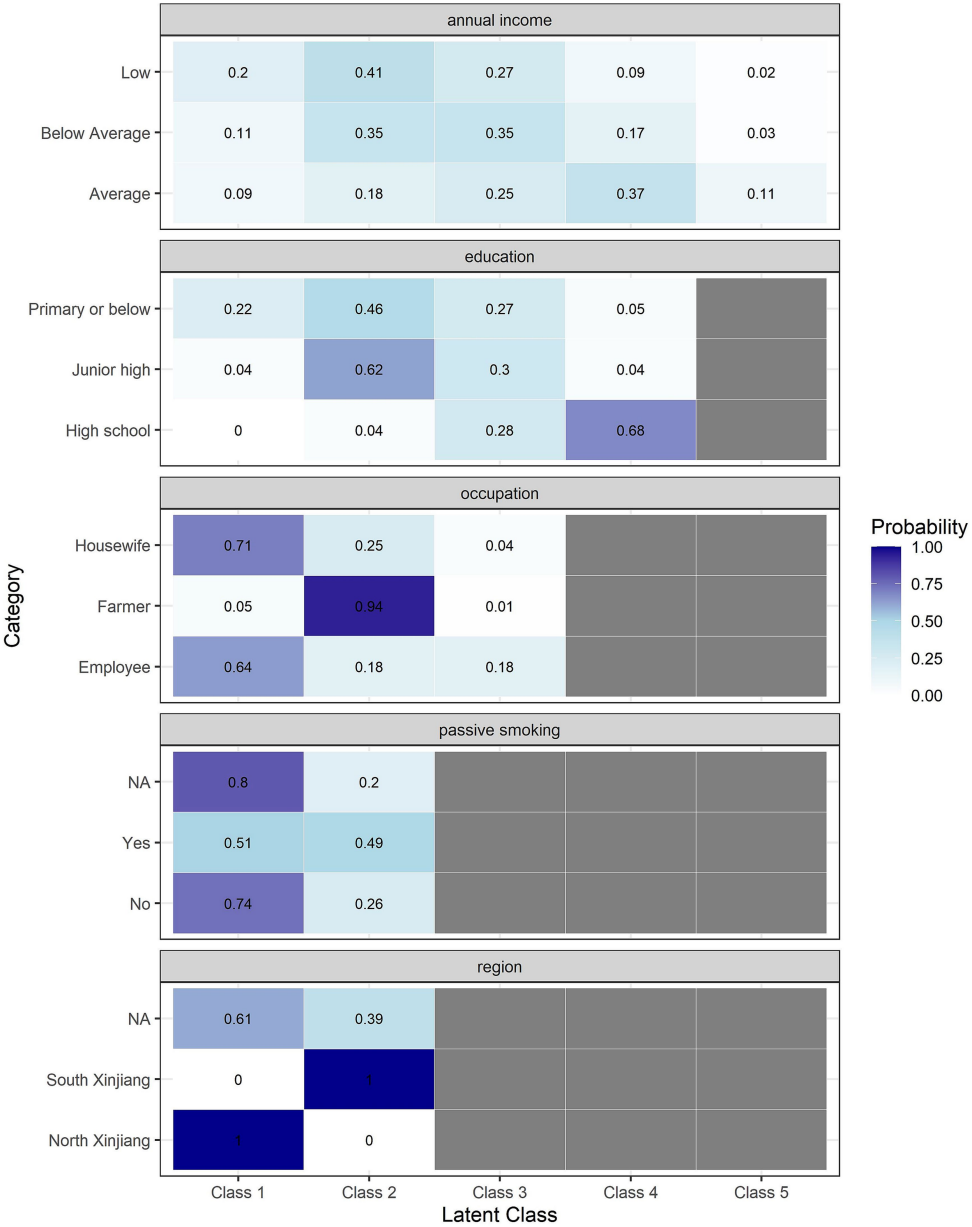
Characteristic profiles of socioeconomic status patterns identified based on latent class analysis.

A total of 1,166 pregnant women were included in this study. The participant recruitment, screening, exclusion, and final inclusion process is shown in Figure 1. To identify potential patterns of SES characteristics among the pregnant women, five indicators—region, annual income, education level, occupation, and passive smoking status—were included in the LCA. The model fit results for the latent class analysis (Supplementary Table S1) indicated that the three-class model was the optimal model (AIC = 11068.41, BIC = 11245.55), with the highest entropy value (0.977), suggesting high classification accuracy and balanced proportions across classes. Based on the conditional probabilities (Figure 2), three distinct socioeconomic and behavioral patterns were identified. Pattern A (37.14%) was defined as the Northern Xinjiang-Housewife-dominant Low SES type, characterized primarily by residence in Northern Xinjiang, low education level, a predominance of housewives, and a higher rate of passive smoking exposure. Pattern B (41.60%) was defined as the Southern Xinjiang-Housewife-dominant Low-to-Middle SES type, characterized primarily by residence in Southern Xinjiang, a predominance of housewives, and low-to-mid levels of education and income, with agricultural workers also represented in this class. Pattern C (21.27%) was defined as the Highly Educated-Professional Woman-High SES type, characterized by distribution across both Southern and Northern Xinjiang, very high education levels and stable professional status, along with the highest household income and the lowest passive smoking rate.

### 3.2 Basic characteristics and thyroid-related indicators of pregnant women across different SES patterns

As shown in Table 1, significant differences (*p* < 0.001) were observed among the three SES patterns regarding age, parity, income, education, occupation, and passive smoking. Regarding iodine nutritional status, significant differences (all *p* < 0.001) were found among the different SES patterns in long-term dietary iodine intake (FFQ), short-term dietary iodine intake (24-h recall), and the UICr. Specifically, Pattern C had the highest estimated iodine intake from FFQ (322.72 μg/d), while Pattern B had the highest UICr level (283.34 μg/g), and Pattern A had the highest short-term dietary iodine intake (288.46 μg/d). Analysis of thyroid function and structural indicators revealed statistically significant differences in FT3, FT4, and TSH levels among the three groups (*p* < 0.05). No significant difference was found in thyroid volume among the groups (*p* = 0.106). However, the prevalence of thyroid nodules differed significantly among the three groups (*p* < 0.001), being highest in Pattern A (21%). Concerning thyroid disorders, the prevalence of subclinical hypothyroidism differed significantly among the groups (*p* < 0.001), with Pattern A having the highest rate (18%). In contrast, no significant differences were observed among the groups in the prevalence of hypothyroxinemia, clinical hyperthyroidism, or subclinical hyperthyroidism.

**Table 1:** Comparison of basic characteristics and thyroid-related indicators among pregnant women across three SES patterns.

Variable	Overall (*N* = 1,166)	Pattern A (*N* = 433)	Pattern B (*N* = 485)	Pattern C (*N* = 248)	*P* value
Age (years)	26.00 (23.00, 30.00)	27.00 (23.00, 31.00)	25.00 (22.00, 28.00)	27.00 (24.00, 30.00)	<0.001
BMI (kg/m^2^)	24.24 (21.97, 26.67)	24.61 (21.87, 27.11)	24.14 (21.97, 26.22)	24.22 (22.04, 26.81)	0.265
Gestational weeks	24.00 (16.00, 31.00)	24.00 (16.00, 32.00)	23.00 (16.00, 31.00)	24.00 (15.00, 31.00)	0.574
Parity					<0.001
Primiparous	573 (49.14%)	176 (40.65%)	237 (48.87%)	160 (65.52%)	
Multiparous	593 (50.86%)	257 (59.35%)	248 (51.13%)	88 (35.48%)	
Annual income					<0.001
Low	159 (13.64%)	91 (21.02%)	51 (10.52%)	17 (6.85%)	
Below average	392 (33.62%)	185 (42.73%)	173 (35.67%)	34 (13.71%)	
Average	350 (30.02%)	108 (24.94%)	167 (34.43%)	75 (30.24%)	
Above average	213 (18.27%)	39 (9.01%)	80 (16.49%)	94 (37.90%)	
High	52 (4.46%)	10 (2.31%)	14 (2.89%)	28 (11.29%)	
Education					<0.001
Primary or below	113 (9.69%)	94 (21.71%)	19 (3.92%)	0 (0.00%)	
Junior high	505 (43.31%)	201 (46.42%)	304 (62.68%)	0 (0.00%)	
High school	332 (28.47%)	120 (27.71%)	145 (29.90%)	67 (27.02%)	
College or above	216 (18.52%)	18 (4.16%)	17 (3.51%)	181 (72.98%)	
Occupation					<0.001
Housewife	622 (53.34%)	306 (70.67%)	310 (63.92%)	6 (2.42%)	
Employee	440 (37.74%)	110 (25.40%)	88 (18.14%)	242 (97.58%)	
Farmer	104 (8.92%)	17 (3.93%)	87 (17.94%)	0 (0.00%)	
Passive smoking	396 (33.96%)	108 (24.94%)	241 (49.69%)	47 (18.95%)	<0.001
FFQ-derived dietary iodine intake (μg/d)	267.03 (209.93, 353.85)	286.45 (233.64, 347.74)	228.17 (188.51, 292.30)	322.72 (243.40, 479.88)	<0.001
3-day 24-h recall-derived dietary iodine intake (μg/d)	228.10 (170.56, 287.95)	288.46 (243.36, 335.76)	173.71 (157.13, 212.21)	233.58 (184.88, 285.43)	<0.001
UICr (μg/g)	234.19 (132.76, 377.46)	199.90 (104.66, 309.76)	283.34 (181.89, 422.23)	211.74 (121.34, 358.38)	<0.001
FT3 (pmol/L)	4.24 (3.84, 4.69)	4.10 (3.68, 4.58)	4.37 (3.91, 4.79)	4.23 (3.88, 4.70)	<0.001
FT4 (pmol/L)	14.03 (12.59, 15.67)	13.63 (12.06, 15.47)	14.18 (12.87, 15.76)	14.12 (12.65, 15.77)	0.003
TSH (mIU/L)	2.51 (1.70, 3.51)	2.80 (1.95, 4.14)	2.26 (1.61, 3.28)	2.48 (1.68, 3.30)	<0.001
Thyroid volume (mL)	6.38 (5.13, 7.88)	6.32 (5.01, 7.68)	6.46 (5.29, 7.77)	6.38 (5.09, 8.13)	0.106
Thyroid nodule	161 (13.81%)	89 (20.55%)	32 (6.60%)	40 (16.13%)	<0.001
TgAb					0.636
Negative	1,100 (94.34%)	409 (94.46%)	460 (94.85%)	231 (93.15%)	
Positive	66 (5.66%)	24 (5.54%)	25 (5.15%)	17 (6.85%)	
TPOAb					0.029
Negative	1,043 (89.45%)	376 (86.84%)	447 (92.16%)	220 (88.71%)	
Positive	123 (10.55%)	57 (13.16%)	38 (7.84%)	28 (11.29%)	
Hypothyroxinemia	42 (3.60%)	14 (3.23%)	19 (3.92%)	9 (3.63%)	0.857
Subclinical hyperthyroidism	8 (0.69%)	0 (0.00%)	5 (1.03%)	3 (1.21%)	0.089
Hyperthyroidism	8 (0.69%)	1 (0.23%)	5 (1.03%)	2 (0.81%)	0.330
Subclinical hypothyroidism	121 (10.38%)	76 (17.55%)	26 (5.36%)	19 (7.66%)	<0.001

Normally distributed continuous variables are presented as mean ± standard deviation; non-normally distributed continuous variables are presented as median (interquartile range); categorical variables are presented as frequency and percentage. In this table, Average dietary iodine intake refers to FFQ-derived iodine intake, which was estimated using the food frequency questionnaire and reflects habitual iodine intake over the previous 3 months. Daily average dietary iodine intake refers to 3-day 24-h recall-derived iodine intake, which was estimated using the retrospective 3-day 24-h dietary recall and reflects recent iodine intake.

### 3.3 Association of SES patterns with thyroid function indices

**Figure 3:**
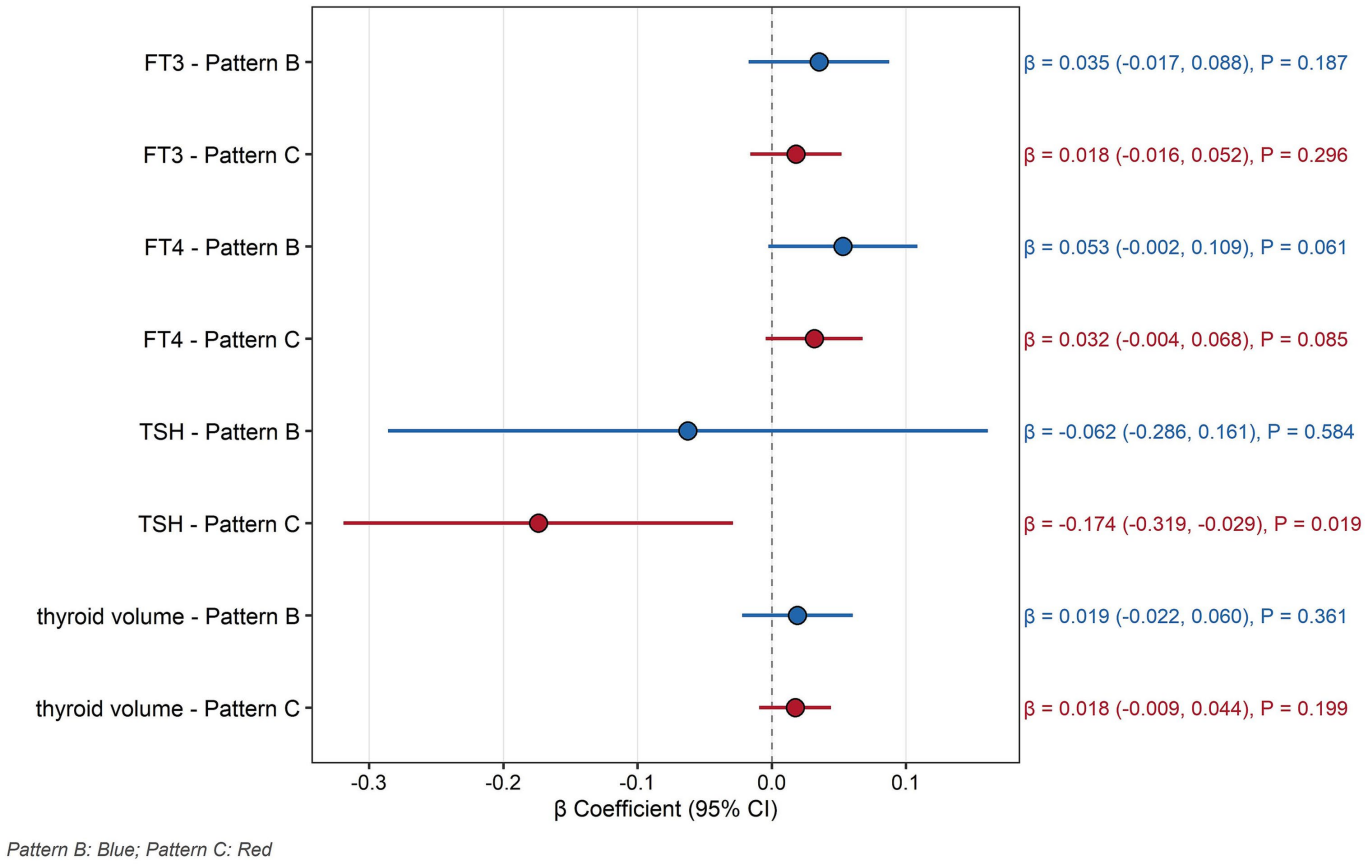
Association of SES patterns with thyroid function indices.

Using Pattern A as the reference group, the results from the multivariable linear regression models, after adjustment for maternal age, gestational weeks, BMI, parity, region, and passive smoking, showed that Pattern C was significantly associated with lower TSH levels compared with Pattern A (*β* = −0.17, 95% CI: −0.32– -0.03, *p* = 0.019) (Figure 3; Supplementary Table S2). No statistically significant association was observed between Pattern B and TSH levels. Regarding free thyroid hormones, neither Pattern B nor Pattern C showed statistically significant associations with FT3 or FT4 levels compared with Pattern A. Similarly, no significant associations were observed between SES patterns and thyroid volume.

### 3.4 Association of SES patterns with thyroid disease risk

**Figure 4:**
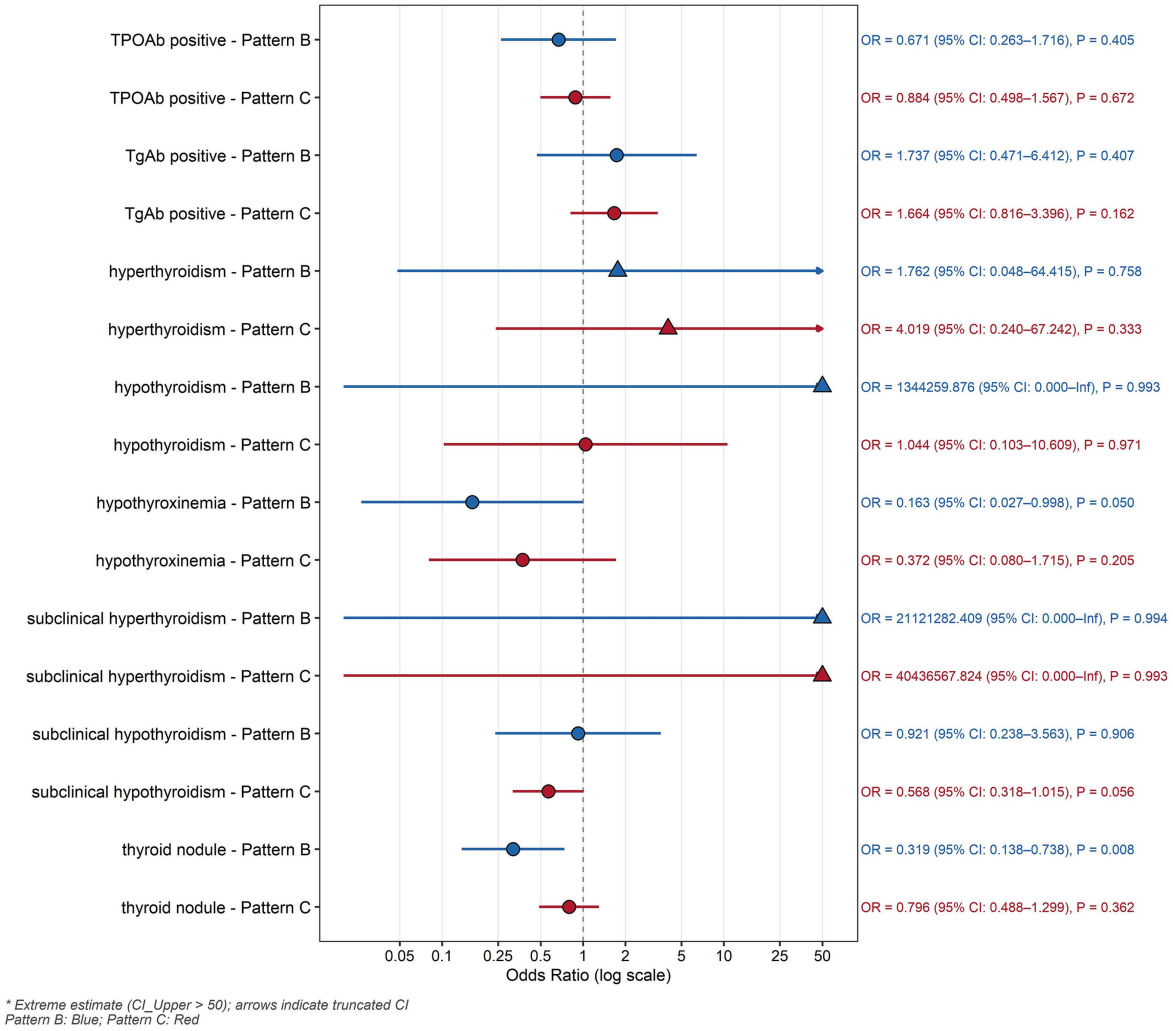
Association of SES patterns with thyroid disease risk.

Using Pattern A as the reference group, the results from the multivariable logistic regression models, after adjustment for maternal age, gestational weeks, BMI, parity, region, and passive smoking, showed that Pattern B was significantly associated with lower odds of thyroid nodules compared with Pattern A (OR = 0.32, 95% CI: 0.14–0.74, *p* = 0.008) (Figure 4; Supplementary Table S3). This corresponds to approximately 68.00% lower odds of thyroid nodules in Pattern B. No other thyroid disease outcomes showed statistically significant associations with SES patterns after adjustment.

### 3.5 Mediation analysis of iodine nutrition

To explore whether the associations between different SES patterns and thyroid-related outcomes were statistically mediated by iodine nutritional indicators, mediation analysis was performed. To ensure the reliability of statistical results, mediation analysis was performed only for thyroid diseases with an event count ≥10. The results (Table 2) showed that across all tested pathways, whether for continuous indices (TSH, FT4, FT3, thyroid volume) or binary disease outcomes (thyroid nodules, subclinical hypothyroidism, TPOAb positivity), none of the three iodine nutrition indicators showed a statistically significant mediation association (all ACME *p* > 0.05). However, the direct associations between SES patterns and TSH, thyroid nodules, and subclinical hypothyroidism remained statistically significant (*p* < 0.05).

**Table 2:** Mediating effects of iodine nutritional status in the association between socioeconomic status patterns and thyroid outcomes.

Outcome variable	Mediating variable	ACME (95% CI)	*P* value	ADE (95% CI)	*P* value
TSH (mIU/L)	Iodine intake (FFQ)	−0.001 (−0.007, 0.007)	0.786	−0.141 (−0.203, −0.075)	<0.001
	Iodine intake (24 h)	−0.001 (−0.016, 0.010)	0.848	−0.141 (−0.203, −0.075)	<0.001
	UICr	0.000 (−0.002, 0.003)	0.498	−0.142 (−0.203, −0.077)	<0.001
FT4 (pmol/L)	Iodine intake (FFQ)	0.000 (−0.002, 0.001)	0.698	0.014 (−0.003, 0.030)	0.086
	Iodine intake (24 h)	0.001 (−0.001, 0.004)	0.452	0.013 (−0.004, 0.029)	0.112
	UICr	0.000 (−0.001, 0.001)	0.464	0.013 (−0.002, 0.029)	0.098
FT3 (pmol/L)	Iodine intake (FFQ)	−0.001 (−0.002, 0.001)	0.346	0.015 (−0.000, 0.031)	0.058
	Iodine intake (24 h)	0.000 (−0.003, 0.003)	0.984	0.015 (−0.001, 0.031)	0.070
	UICr	0.000 (−0.001, 0.001)	0.504	0.015 (−0.001, 0.031)	0.060
Thyroid volume (mL)	Iodine intake (FFQ)	0.000 (−0.001, 0.001)	0.604	0.014 (0.000, 0.028)	0.050
	Iodine intake (24 h)	0.000 (−0.001, 0.001)	0.436	0.015 (0.001, 0.029)	0.044
	UICr	0.000 (−0.001, 0.000)	0.986	0.014 (0.001, 0.028)	0.044
Thyroid nodule	Iodine intake (FFQ)	0.002 (0.000, 0.007)	0.128	−0.037 (−0.072, −0.007)	0.012
	Iodine intake (24 h)	−0.001 (−0.006, 0.002)	0.514	−0.033 (−0.070, −0.003)	0.028
	UICr	0.000 (−0.002, 0.002)	0.404	−0.034 (−0.071, −0.004)	0.028
Subclinical hypothyroidism	Iodine intake (FFQ)	0.002 (−0.002, 0.006)	0.278	−0.064 (−0.098, −0.034)	<0.001
	Iodine intake (24 h)	−0.002 (−0.007, 0.002)	0.330	−0.062 (−0.097, −0.031)	<0.001
	UICr	0.000 (−0.003, 0.001)	0.436	−0.063 (−0.097, −0.032)	<0.001
Hypothyroxinemia	Iodine intake (FFQ)	0.000 (−0.002, 0.001)	0.880	0.001 (−0.015, 0.014)	0.876
	Iodine intake (24 h)	0.000 (−0.002, 0.005)	0.924	0.001 (−0.014, 0.013)	0.936
	UICr	0.000 (−0.003, 0.000)	0.232	0.001 (−0.016, 0.019)	0.902
TPOAb positive	Iodine intake (FFQ)	0.000 (−0.003, 0.002)	0.854	−0.013 (−0.039, 0.011)	0.296
	Iodine intake (24 h)	0.000 (−0.004, 0.003)	0.794	−0.012 (−0.039, 0.012)	0.298
	UICr	0.000 (0.000, 0.003)	0.850	−0.013 (−0.040, 0.011)	0.266
TgAb positive	Iodine intake (FFQ)	0.000 (−0.002, 0.001)	0.596	0.009 (−0.008, 0.023)	0.334
	Iodine intake (24 h)	0.002 (0.000, 0.006)	0.070	0.006 (−0.012, 0.020)	0.508
	UICr	0.000 (−0.002, 0.001)	0.680	0.008 (−0.009, 0.023)	0.328

ACME, average causal mediation effect; ADE, average direct effect; CI, confidence interval; FFQ, food frequency questionnaire; UICr, urinary iodine-to-creatinine ratio; TSH, thyroid-stimulating hormone; FT3, free triiodothyronine; FT4, free thyroxine; TgAb, thyroglobulin antibody; TPOAb, thyroid peroxidase antibody. Mediation analyses were performed using adjusted mediator and outcome models, controlling for maternal age, gestational week, BMI at enrollment, and parity. Linear regression models were used for continuous outcomes, and Probit regression models were used for binary outcomes. *P* values were calculated using quasi-Bayesian Monte Carlo simulations with 1,000 iterations.

## 4 Discussion

This study employed latent class analysis to identify three distinct SES patterns among pregnant women in Xinjiang and examined their associations with thyroid-related indicators. After adjustment for potential confounders, Pattern C was associated with lower TSH levels compared with Pattern A, whereas Pattern B was associated with lower odds of thyroid nodules. However, most other thyroid functional and disease outcomes were not significantly associated with SES patterns after adjustment. Exploratory mediation analysis suggested that dietary and urinary iodine indicators did not statistically mediate the associations between SES patterns and thyroid-related outcomes.

Previous epidemiological studies have often treated low income or low education as uniform risk factors for thyroid disease, positing that resource scarcity leads to health disadvantages (26–28). However, this study, through LCA, revealed significant health inequalities within the low SES population. Pattern A (Northern Xinjiang-Housewife-Low SES) was the subgroup with the poorest thyroid health in this study, exhibiting a subclinical hypothyroidism prevalence as high as 17.55%, significantly higher than the other two groups. This phenomenon appears paradoxical given Northern Xinjiang’s relatively superior regional economic level. This may stem from the combined effects of psychosocial stress from relative deprivation and indoor environmental exposures (29, 30). First, the disparity between regional economic advantage and individual social status may exacerbate psychosocial stress (31, 32). Although Northern Xinjiang has a higher overall level of economic development, Pattern A women were at the bottom in terms of both education and income. This contrast between an affluent objective environment and scarce individual resources creates a strong sense of relative deprivation. Compared to low-income individuals in an overall poor environment, those of low status situated within a wealthy environment often face more severe social comparison pressure and psychological distress (33). Prolonged exposure to this situation of social status inconsistency subjects individuals to chronic psychosocial stress, leading to increased allostatic load (34, 35). Long-term abnormal fluctuations in cortisol levels can inhibit thyroid-stimulating hormone secretion via neuroendocrine pathways and interfere with thyroid hormone synthesis and conversion, thereby increasing the risk of hypothyroidism (36). Second, the specific geographical and climatic characteristics of Northern Xinjiang, combined with the occupational exposures of housewives, create a unique risk superposition of indoor environmental hazards. Northern Xinjiang has long, cold winters, leading residents to reduce window ventilation for warmth, resulting in significantly longer indoor activity times compared to Southern Xinjiang (37). As low-educated housewives, this group not only undertakes primary cooking duties long-term but also faces a high rate of secondhand smoke exposure. In poorly ventilated, confined indoor environments, polycyclic aromatic hydrocarbons released from cooking fumes and thiocyanates from secondhand smoke tend to accumulate (38, 39). Thiocyanate acts as a competitive inhibitor blocking thyroidal iodine uptake, while polycyclic aromatic hydrocarbons have established thyroid toxicity (40). Importantly, our multivariable analysis confirmed that the association of Pattern A with subclinical hypothyroidism remained highly significant even after statistically adjusting for passive smoking exposure, suggesting a multi-layered toxicological and social burden on this group. Compared to women in Southern Xinjiang engaged in agricultural labor in open environments, the Pattern A group not only lacks regular physical activity but is also chronically exposed to this complex mixture of indoor chemical pollutants. Therefore, specific environmental toxicological mechanisms, combined with a sedentary lifestyle, constitute the unique thyroid health risk profile for this subgroup.

In contrast, Pattern B (Southern Xinjiang-Housewife-dominant Low-to-Middle SES), despite similarly low income and education levels and the highest rate of secondhand smoke exposure (49.69%), was associated with lower odds of thyroid nodules compared with Pattern A. This finding suggests that low SES should not be simplistically equated with uniformly higher thyroid health risk, and that specific social roles, regional context, and lifestyle patterns may also be important. Although Pattern B was housewife-dominant, agricultural workers were also represented in this class, and women in southern Xinjiang may have different daily activity patterns, outdoor exposure, dietary habits, and community environments compared with those in northern Xinjiang. These contextual differences may partly explain the lower prevalence of thyroid nodules observed in Pattern B. First, differences in physical activity and lifestyle patterns may be relevant. Compared with women in Pattern A, who were mainly housewives in northern Xinjiang, women in Pattern B may have been exposed to more outdoor activities or household and agricultural-related physical work. Previous studies have suggested that regular physical activity may improve chronic low-grade inflammatory status and metabolic balance by downregulating pro-inflammatory factors (41, 42). These mechanisms may be related to thyroid structural abnormalities, although the present cross-sectional study cannot establish causality. Second, the social environment in southern Xinjiang may also play a role. Rural and semi-rural communities often retain stronger neighborhood and family support networks, which may provide pregnant women with emotional support and practical assistance (43, 44). Such social support may help buffer stress associated with material disadvantage. Therefore, the relatively lower odds of thyroid nodules observed in Pattern B may reflect the combined influence of regional lifestyle, social context, and unmeasured environmental or genetic factors, rather than income or education alone.

Pattern C (Highly Educated-Professional Female-High SES) demonstrated typical health advantages, particularly evidenced by the superior stability of thyroid function indicators. Quantitative assessment using the coefficient of variation (CV) substantiated this finding, as Pattern C exhibited the lowest TSH variability (57.78%) compared to Pattern A (61.42%) and Pattern B (70.39%). Furthermore, Levene’s test confirmed that the differences in TSH variability across the three patterns were statistically highly significant (*p* < 0.001; Supplementary Table S4), indicating more robust homeostatic control in this group. This aligns with the fundamental cause theory, which posits that high SES individuals possess more flexible resources such as knowledge, wealth, and social networks, enabling them to proactively avoid health risks (45, 46). Additionally, this group’s lower rate of secondhand smoke exposure (18.95%) reflects their ability to utilize social resources to actively construct smoke-free environments. This proactive management of environmental toxin exposure further consolidates their health advantage. Notably, although this group had the highest estimated dietary iodine intake, their UICr as controlled within the optimal range without risk of iodine excess. This suggests that this group likely possesses higher health literacy, enabling more scientific balancing of dietary nutrition and avoiding side effects from indiscriminate iodine supplementation (47). Nevertheless, the prevalence of thyroid nodules in Pattern C (16.13%) was higher than that in Pattern B (6.60%). This finding is potentially associated with surveillance bias, as high SES individuals generally have greater access to healthcare services and are more likely to undergo routine neck ultrasounds during annual physical examinations (48). Given that thyroid nodules are frequently asymptomatic, the elevated rate in Pattern C may indicate higher diagnostic capture rather than a more severe underlying pathology. Additionally, the occupational stress associated with professional roles in this group may serve as a contributing factor, necessitating further study.

Although statistically significant differences in dietary iodine intake were observed among pregnant women of different SES patterns, mediation effect analysis strongly confirmed that iodine nutritional status was not a mediating pathway through which SES leads to differences in thyroid disease risk. This negative finding has important public health implications. It suggests that in a context where iodine deficiency disorders have been fundamentally eliminated and the overall population’s iodine nutrition is at an adequate level, minor fluctuations in iodine intake are no longer the dominant force driving thyroid health inequalities (49). Nutritional intervention strategies relying solely on iodine supplementation or restriction may be insufficient to effectively eliminate the disease burden arising from socioeconomic disparities (50). After excluding the mediating role of iodine nutrition, the significant direct effect of SES on thyroid health suggests the existence of other, more concealed biopsychosocial influences. This health disparity independent of nutrition is more likely attributable to the specific levels of psychosocial stress, differential exposure to environmental endocrine disruptors, and metabolic effects of lifestyle associated with different SES patterns (51, 52). Therefore, the results of this study have certain guiding significance for formulating thyroid disease prevention and control strategies. Given that the driving effect of socioeconomic status on thyroid disease risk is independent of iodine nutritional status, current perinatal healthcare strategies focusing solely on iodine nutrition monitoring may be insufficient. Public health interventions should expand from single nutritional supplementation to comprehensive management of high-risk social environments. Particularly for urban housewife groups with low social status and lack of social support, mental health interventions and indoor environmental hygiene guidance should be strengthened to block disease occurrence at the level of social etiology.

This study has several strengths. First, instead of using traditional single indicators to measure socioeconomic status, latent class analysis was applied to handle multidimensional socioeconomic variables. This method overcomes the limitation of single indicators in capturing complex social stratification, thereby more accurately depicting the true socioeconomic profiles of pregnant women in Xinjiang’s multi-ethnic region and providing a more reliable classification basis for identifying high-risk subgroups. Second, regarding iodine nutrition assessment, this study integrated a food frequency questionnaire reflecting long-term intake patterns, a 3-day 24-h dietary recall reflecting recent intake, and urinary iodine test results. This multi-dimensional assessment strategy greatly improved the accuracy of iodine nutrition exposure measurement, ensuring the robustness of the mediation analysis conclusion and strongly supporting the core finding that iodine is not the primary factor.

However, this study also has several limitations. First, because of the cross-sectional design, the temporal and causal relationships among socioeconomic status, iodine nutritional indicators, and thyroid health outcomes cannot be established, although mediation analysis was used to explore potential statistical pathways. Therefore, the findings should be interpreted as associations rather than evidence of causality. Second, although the analytical models adjusted for several potential confounders, including age, gestational week, BMI, and parity, some important factors were not fully measured or adjusted for. For example, this study lacked direct measurements of psychological stress, environmental endocrine disruptor exposure, active smoking history, and alcohol consumption history (53, 54). In addition, Xinjiang is characterized by substantial ethnic diversity, and ethnic composition as well as genetic background may differ between northern and southern Xinjiang. These factors may be associated with both socioeconomic patterns and thyroid-related outcomes and may therefore have influenced the observed associations. As such, residual confounding cannot be completely excluded. Future studies should incorporate more detailed assessments of ethnicity, genetic susceptibility, psychosocial stress, environmental exposures, and lifestyle factors to further clarify the associations between social factors and thyroid health. Third, considering Xinjiang’s vast territory and complex ethnic composition, and because the study sample was primarily drawn from two representative regions in northern and southern Xinjiang, caution is warranted when generalizing the findings to other regions or subgroups across Xinjiang.

## 5 Conclusion

This study found that in Xinjiang, in the context of eliminated iodine deficiency disorders, socioeconomic patterns are important independent predictors of thyroid disease risk in pregnant women, and this influence is not dependent on differences in iodine intake. This finding suggests that future strategies for promoting perinatal thyroid health should move beyond single nutritional intervention models towards more precise social stratification interventions. Future public health practices should focus on housewife groups with lower socioeconomic status, employing multi-faceted approaches such as strengthening psychosocial support, healthy lifestyle guidance, and indoor environmental management to block disease occurrence at the source of social etiology, thereby promoting equity in regional maternal and child health.

## Data Availability

The data involved in this study are not publicly available due to privacy but are available from the authors on reasonable request. Requests to access the datasets should be directed to CW, 357935099@qq.com.
