# Supplementary material for: Beyond iodine nutrition: socioeconomic patterns independently drive thyroid disease risk in pregnant women in Xinjiang
Source: Front Nutr. 2026 Jun 19;13:1789303. doi: 10.3389/fnut.2026.1789303 (PMC13327873; doi:10.3389/fnut.2026.1789303)
Supplement: Supplementary file 1 [file Table_1.docx]

Supplementary Material

# Supplementary Tables

**Table S1.** Model Fit Results of Latent Class Analysis

| Classes | LogLik | AIC | BIC | Entropy | Min Class Size | Percent Min Class |
| --- | --- | --- | --- | --- | --- | --- |
| 2 | -5598.443 | 11242.886 | 11359.297 | 0.971 | 316 | 27.101 |
| 3 | -5499.203 | 11068.406 | 11245.553 | 0.977 | 248 | 21.269 |
| 4 | -5488.514 | 11071.028 | 11308.911 | 0.956 | 57 | 4.889 |
| 5 | -5476.126 | 11070.252 | 11368.871 | 0.94 | 114 | 9.777 |

**Table S2**. Associations of SES patterns with continuous thyroid-related indicators

| Outcome | Comparison | *β* | SE | 95% CI | *P* value |
| --- | --- | --- | --- | --- | --- |
| FT3 | Pattern B vs Pattern A | 0.035 | 0.027 | -0.017 to 0.088 | 0.187 |
| FT3 | Pattern C vs Pattern A | 0.018 | 0.017 | -0.016 to 0.052 | 0.296 |
| FT4 | Pattern B vs Pattern A | 0.053 | 0.028 | -0.002 to 0.109 | 0.061 |
| FT4 | Pattern C vs Pattern A | 0.032 | 0.018 | -0.004 to 0.068 | 0.085 |
| TSH | Pattern B vs Pattern A | -0.062 | 0.114 | -0.286 to 0.161 | 0.584 |
| TSH | Pattern C vs Pattern A | -0.174 | 0.074 | -0.319 to -0.029 | 0.019 |
| Thyroid volume | Pattern B vs Pattern A | 0.019 | 0.021 | -0.022 to 0.060 | 0.361 |
| Thyroid volume | Pattern C vs Pattern A | 0.018 | 0.014 | -0.009 to 0.044 | 0.199 |

Note: Pattern A was used as the reference group. *β* values and 95% confidence intervals were derived from multivariable linear regression models. Models were adjusted for maternal age, gestational weeks, BMI, parity, region, and passive smoking.

**Table S3**. Associations of SES patterns with thyroid disease outcomes

| Outcome | Comparison | OR | 95% CI | *P* value |
| --- | --- | --- | --- | --- |
| Thyroid nodule | Pattern B vs Pattern A | 0.319 | 0.138 - 0.738 | 0.008 |
| Thyroid nodule | Pattern C vs Pattern A | 0.796 | 0.488 - 1.299 | 0.362 |
| TgAb positivity | Pattern B vs Pattern A | 1.737 | 0.471 - 6.412 | 0.407 |
| TgAb positivity | Pattern C vs Pattern A | 1.664 | 0.816 - 3.396 | 0.162 |
| TPOAb positivity | Pattern B vs Pattern A | 0.671 | 0.263 - 1.716 | 0.405 |
| TPOAb positivity | Pattern C vs Pattern A | 0.884 | 0.498 - 1.567 | 0.672 |
| Isolated hypothyroxinemia | Pattern B vs Pattern A | 0.163 | 0.027 - 0.998 | 0.050 |
| Isolated hypothyroxinemia | Pattern C vs Pattern A | 0.372 | 0.080 - 1.715 | 0.205 |
| Subclinical hyperthyroidism | Pattern B vs Pattern A | NE | NE | 0.994 |
| Subclinical hyperthyroidism | Pattern C vs Pattern A | NE | NE | 0.993 |
| Clinical hyperthyroidism | Pattern B vs Pattern A | 1.762 | 0.048 - 64.415 | 0.758 |
| Clinical hyperthyroidism | Pattern C vs Pattern A | 4.019 | 0.240 - 67.242 | 0.333 |
| Subclinical hypothyroidism | Pattern B vs Pattern A | 0.921 | 0.238 - 3.563 | 0.906 |
| Subclinical hypothyroidism | Pattern C vs Pattern A | 0.568 | 0.318 - 1.015 | 0.056 |
| Clinical hypothyroidism | Pattern B vs Pattern A | NE | NE | 0.993 |
| Clinical hypothyroidism | Pattern C vs Pattern A | 1.044 | 0.103 - 10.609 | 0.971 |

Note: Pattern A was used as the reference group. OR values and 95% confidence intervals were derived from multivariable logistic regression models. Models were adjusted for maternal age, gestational weeks, BMI, parity, region, and passive smoking. NE, not estimable because of sparse data or unstable confidence interval estimation; TgAb, thyroglobulin antibody; TPOAb, thyroid peroxidase antibody.

**Table S4**. Quantitative assessment of thyroid function stability across SES patterns using the coefficient of variation (CV) and Levene’s test.

| Indicator | Pattern A | Pattern B | Pattern C | Levene’s Test |
| --- | --- | --- | --- | --- |
| TSH (CV, %) | 61.42% | 70.39% | 57.78% | F = 6.95, *P* < 0.001 |
| FT4 (CV, %) | 12.35% | 12.44% | 12.59% | - |
| FT3 (CV, %) | 10.96% | 11.23% | 12.72% | - |

Notes: CV (Coefficient of Variation) = (Standard Deviation / Mean) × 100%. A lower CV indicates higher stability. Levene’s test was performed to compare the equality of variances for TSH across the three groups, which is the most sensitive marker for thyroid homeostasis. Bold values indicate the lowest variability (highest stability) among the groups.

**Table S5** Sensitivity Analysis: Model Fit Results of the Four-Indicator LCA Model Excluding Passive Smoking

| Classes | LogLik | AIC | BIC | Entropy | Min Class Size | Percent Min Class |
| --- | --- | --- | --- | --- | --- | --- |
| 2 | -4863.487 | 9768.974 | 9875.262 | 0.68 | 343 | 29.417 |
| 3 | -4788.231 | 9640.461 | 9802.424 | 0.784 | 247 | 21.184 |
| 4 | -4777.89 | 9641.781 | 9859.418 | 0.725 | 188 | 16.123 |
| 5 | -4767.967 | 9643.934 | 9917.246 | 0.713 | 125 | 10.72 |
